# Development of Polyoxymethylene Particles via the Solution-Dissolution Process and Application to the Powder Bed Fusion of Polymers

**DOI:** 10.3390/ma13071535

**Published:** 2020-03-27

**Authors:** Maximilian A. Dechet, Ina Baumeister, Jochen Schmidt

**Affiliations:** 1 Institute of Particle Technology, Friedrich-Alexander-Universität Erlangen-Nürnberg, Cauerstraße 4, D-91058 Erlangen, Germany; maximilian.dechet@fau.de (M.A.D.); ina.baumeister@fau.de (I.B.); 2 Interdisciplinary Center for Functional Particle Systems, Friedrich-Alexander-Universität Erlangen-Nürnberg, Haberstraße 9a, D-91058 Erlangen, Germany

**Keywords:** polyoxymethylene (POM), powder bed fusion, additive manufacturing, precipitation, solution-dissolution, particle analysis

## Abstract

In this study, the development of a polyoxymethylene (POM) feedstock material for the powder bed fusion (PBF) of polymers is outlined. POM particles are obtained via liquid-liquid phase separation (LLPS) and precipitation, also known as the solution-dissolution process. In order to identify suitable POM solvent systems for LLPS and precipitation, in the first step, a solvent screening based on solubility parameters was performed, and acetophenone and triacetin were identified as the most promising suitable moderate solvents for POM. Cloud point curves were measured for both solvents to derive suitable temperature profiles and polymer concentrations for the solution-dissolution process. In the next step, important process parameters, namely POM concentration and stirring conditions, were studied to elucidate their effect on the product’s properties. The product particles obtained from both aforementioned solvents were characterized with regard to their morphology and size distribution, as well as their thermal properties (cf. the PBF processing window) and compared to a cryo-milled POM PBF feedstock. Both solvents allowed for precipitation of POM particles of an appropriate size distribution for PBF for polymer concentrations of at least up to 20 wt.%. Finally, a larger powder batch for application in the PBF process was produced by precipitation from the preferred solvent acetophenone. This POM powder was further analyzed concerning its flowability, Hausner ratio, and mass-specific surface area. Finally, test specimens, namely a complex gyroid body and a detailed ornament, were successfully manufactured from this feedstock powder showing appropriate bulk solid and thermal properties to demonstrate PBF processability. In summary, a processable and suitable POM PBF feedstock could be developed based on the non-mechanical solution dissolution process, which, to the authors’ best knowledge, has not been reported in previous studies.

## 1. Introduction

The term additive manufacturing (AM) is comprised of different technologies, which all have in common that the parts are built layer-by-layer, in contrast to, e.g., “traditional” subtractive manufacturing methods [1], although the technologies themselves vary greatly with respect to the underlying processes and materials used. AM with metals, polymers, ceramics, and composites thereof is possible. In the field of polymer AM, extrusion based processes like fused filament fabrication (FFF) [2], photopolymerization based processes like stereolithography (SLA), and powder based processes like binder jetting (BJ) or powder bed fusion (PBF) are well known [3]. The differences in the underlying mechanisms of these processes are accompanied by fundamental differences in part properties, especially concerning mechanical properties [4]. The most widely applied technology in polymer AM, when functional parts of excellent mechanical properties are desired, is PBF, also known as selective laser sintering (SLS) [5]. In PBF, first, a homogeneous layer of feedstock powder is spread onto a building platform. Then, a laser selectively fuses the polymer material according to the cross-section of the part to be printed in this layer. Subsequently, the building platform is lowered by the height of one layer, and new powder is spread. The process is repeated until the building job is finished. The surrounding non-fused powder acts as a support structure, making complex geometries and undercuts easily accessible by means of PBF. However, these advantages come with some drawbacks, as the requirements on the powder feedstock material used for PBF are very demanding [6]. Not only the polymer material properties like the thermal processing window [7], isothermal crystallization [8], and melt viscosities [9,10], but also the bulk solid properties like packing density, flowability, size distribution, and shape [11], as well as the optical properties [12] need to be optimized. For each PBF material, appropriate PBF process parameters need to be identified to assure a stable manufacturing process and reproducible part properties, i.e., e.g., dimensional accuracy and mechanical strength. Due to the aforementioned demands on PBF polymer feedstocks, despite the evolving research on AM feedstock materials that could be recognized in recent years, the choice of available PBF polymer powders still is very limited. Very few types of PBF feedstock powders are commercially available so far, and the market is still dominated by polyamides, especially polyamide 12 (PA12) [13], making up at least 90 % of the total market share, of which the largest fraction is produced via a solution-dissolution process [14,15]. Other commercially available materials like polyaryl ether ketones (PAEK), polypropylene (PP), or thermoplastic elastomers (TPE) are used to a much lesser degree, as they either demand special manufacturing systems (for high-temperature thermoplasts, PAEK) and/or often show inferior processability compared to PA12 [3]. The current focus on PA12 feedstock materials leads to the limitations of the possible applications of PBF manufactured parts, as there are other polymers that are, e.g., better suited for high temperatures, corrosive environments, biocompatibility, gliding applications, or high mechanical loads. Various viable attempts have been reported in the literature for the production of PBF-suited feedstocks. For example, a process chain [16] employing wet grinding of polymers followed by thermal rounding in a heated downer reactor [17] was proposed to produce PBF powders from thermoplastic granules. Moreover, cryogenic milling [18,19], a melt emulsification process [20], co-extrusion [21], spray agglomeration, spray drying [22], or precipitation based processes [23,24,25] have been reported. Among the various researched materials for PBF are, e.g., polypropylene [26,27], polyethylene [28], polystyrene [29], polybutylene terephthalate [21], PAEKs [11,30,31], polyphenylene sulfide [32], and polylactide [19,25], to only name a few. Despite the good mechanical properties of POM, its high stiffness, high creep resistance, intrinsic whiteness, and low coefficient of friction, reports on PBF of POM are scarce, with only a patent application for POM PBF powders [33] and publications by Rietzel et al. [34,35]. In the aforementioned studies, the powder was manufactured via cryogenic milling and, thus, suffered from the known issues with milled PBF feedstocks, like an unfavorable particle shape and size distribution or inferior flowability and spreadability. Consequently, the manufacturing of well-suited POM feedstocks for PBF should remain of high interest to PBF users. In this study, we showcase a non-mechanical approach, based on the solution-dissolution process, to obtain POM powders applicable to PBF. To the best of the authors’ knowledge, this is the first report on the application of the solution-dissolution process for the production of POM PBF feedstocks in the open literature. The processability of this feedstock is proven by manufacturing multi-layered specimens.

## 2. Materials and Methods

### 2.1. Materials

Injection-grade polyoxymethylene copolymer (Hostaform C52021, Celanese Co., Dallas, TX, USA) granules, with a degree of crystallinity of 55%, as determined via density (see Section 2.3.7), were used as the feed material for the solution-dissolution process. The solvents used for screening were acetophenone (99%, Alfa Aesar, Ward Hill, MA, USA), triacetin (99%, Alfa Aesar, Ward Hill, MA, USA), benzaldehyde (≥99.5%, Carl Roth, Karlsruhe, Germany), benzyl benzoate (99%, Alfa Aesar, Ward Hill, MA, USA), benzyl ether (98%, Sigma-Aldrich, St. Louis, MO, USA), and diethylene glycol n-butyl ether acetate (DEGBEA) (≥97%, Carl Roth, Karlsruhe, Germany). Technical ethanol (96%, VWR, Radnor, PA, USA) was used as the washing solvent. All solvents were used without further purification.

### 2.2. Experimental

#### 2.2.1. Solution-Dissolution Process

Solvent-screening and powder production experiments were conducted in autoclaves (DAB-3, Berghof, Eningen, Germany), with a maximum volume of 250 mL. Screening experiments were conducted with 10 wt.% POM in each solvent, while the total batch size (weight of polymer and solvent) in each experiment was 100 g. Heating of the autoclaves was realized by magnetic stirring hot plates equipped with aluminum heating blocks. Thermocouples (Type K), placed inside the aluminum block, were used to monitor the process temperature. PTFE-coated magnetic bars of 6 mm in diameter and 25 mm in length were used for stirring. For screening, the autoclaves were heated to 190 °C, and they were kept for 15 min at this temperature to ensure complete dissolution of the POM. The stirring rate for the screening experiments during heating and cooling was kept constant at 100 rpm. For all experiments, the autoclaves were allowed to cool down to at least 60 °C, resulting in rather small cooling rates in the range of 0.4 to 3 K/min. The obtained suspensions were washed with ethanol, and the precipitated solid was collected by filtration using a Büchner funnel. The obtained particles were dried in a ventilated oven for at least 48 h at 55 °C. Comprehensive information on the mechanism of the solution-dissolution process, the theoretical background, and the correlation of process parameters and product properties can be found elsewhere [36,37,38,39,40,41,42].

#### 2.2.2. Optical Cloud Point Determination

The cloud point curves for POM in the favored solvents triacetin and acetophenone, as identified in the solvent screening experiments, were determined visually. Solution temperatures dependent on the polymer concentration were determined for up to 30 wt.% POM, according to a procedure outlined in [23,25]: total batch sizes of 100 g were heated in a beaker, equipped with Type K thermocouples, on a hot plate stirrer under constant magnetic stirring until a clear solution was observed. After holding the system in an isothermal condition for 15 min in the state of a clear solution, the heating was turned off, and the system was allowed to cool down. The cloud point for the investigated system composition was defined as the temperature at which turbidity could be observed visually. The reported cloud points were the mean values obtained from two experiments. For the first heating cycle, the temperature at which complete dissolution of the POM granules was observable was disregarded, as the size of the granules and the mixing conditions [43] distorted a clear dissolution range. Thus, the determination of a distinct dissolution temperature was impractical. However, the temperature of complete dissolution of the formed small POM particles could be well observed for the second cycle.

#### 2.2.3. Powder Bed Fusion of Polymers

To investigate the PBF processability of the precipitated POM powder, test specimens were manufactured on a desktop PBF machine (SnowWhite, Sharebot, Nibionno, Italy). The device was equipped with a CO_2_ laser (wavelength 10.6 µm) of a maximum nominal power of 14 W and allowed for a maximum scanning speed of 3500 mm/s. The desktop sized SnowWhite device, which was operated under ambient air in the building chamber, did not yield parts with mechanical properties comparable to those of parts produced with industrial PBF machines under an inert building chamber atmosphere and optimized parameters. As outlined in [25], tensile test specimens manufactured from standard PA12 PBF feedstock (PA2200, EOS, Krailling, Germany) with the parameters recommended by the SnowWhite manufacturer exhibited a much lower ultimate tensile strength compared to specimens built in an EOS machine. Nevertheless, the desktop device allowed for profound qualitative powder assessment with respect to basic PBF processability and was a useful tool in PBF feedstock development due to the low amount of powder required. Additionally, it was one of the few desktop PBF devices equipped with a CO_2_ laser, which typically is also used in industrial PBF machines. The test specimens were a modified version of the “gyroid cylinder” [44], uploaded by the user “mechadense” to the Thingiverse platform under the Creative Commons—Public Domain Dedication license and an ornament uploaded by the user “3d-decoratie” to the Thingiverse platform [45] under the Creative Commons—Attribution—Share Alike license.

### 2.3. Characterization

#### 2.3.1. Scanning Electron Microscopy

Images of the particle shape and surface morphology were obtained via scanning electron microscopy (SEM) using a GeminiSEM 500 (Carl Zeiss AG, Oberkochen, Germany) operated at an acceleration voltage of 1.0 kV, which was equipped with a secondary electron (SE2) detector.

#### 2.3.2. X-ray Diffraction

X-ray diffraction (XRD) was performed with an AXS D8 Advance diffractometer in Bragg–Brentano geometry (Bruker, Billerica, MA, USA) equipped with a VANTEC-1 detector and a Ni filter; Cu Kα-radiation (154 pm) was used. The powder samples were prepared in PMMA sample holders. Diffractograms were collected at a step size of 0.014° and a measuring time of 1 s per step in the range of 10° ≤ 2θ ≤ 60°.

#### 2.3.3. Laser Diffraction Particle Sizing

In order to measure the particle size distribution (PSD) of the product particles, laser diffraction particle sizing with a Mastersizer 2000 equipped with a Scirocco 2000 dry dispersion unit (Malvern Panalytical GmbH, Kassel, Germany) was used. The dispersion gas (compressed air) pressure was 2 bar. The given PSDs were the average over 3 single determinations.

#### 2.3.4. Flowability

Powder flowability was assessed by shear experiments using a Schulze ring shear tester RST 01.01 (Dr. Dietmar Schulze Schüttgutmesstechnik, Wolfenbüttel, Germany). In the measurements, the unconfined yield strength σ_c_ was determined as a function of the major consolidation stress σ_1_. The experiments were carried out similarly to the procedure described by Schulze [46], with applied consolidation stresses σ_1_ of about 1200 Pa, 2400 Pa, and 4600 Pa. The ratio of consolidation stress σ_1_ and unconfined yield strength σ_c_ was the flow function ff_c_, which allowed for the classification of powder flowability according to Jenike [47]. Furthermore, the Hausner ratio, given by the quotient of powder tapped density and powder bulk density, was evaluated as an indicator of powder flowability. The given Hausner ratio was the mean over 5 experiments.

#### 2.3.5. Nitrogen Sorption Measurements

Nitrogen sorption experiments at liquid nitrogen temperature (77 K) were performed using a volumetric gas sorption analyzer Nova4200e (Quantachrome, Odelzhausen, Germany). Degassing of the samples prior to measurement was performed under vacuum conditions for at least 1 h at 80 °C. The mass-specific BET surface area [48] was determined by a 5-point determination in the relative pressure range $0.05<\frac{p}{p_{0}}<0.3$ (with p: chosen (equilibrium) pressure in the measurement cell, p_0_: reference (ambient) pressure).

#### 2.3.6. Differential Scanning Calorimetry

Thermal characterization of the manufactured POM powders was performed via differential scanning calorimetry (DSC). The thermograms were measured in the temperature range from 30 °C to 180 °C at a heating rate of 10 K/min under a constant nitrogen flow of 25 mL/min using a DSC8000 (Perkin Elmer, Waltham, MA, USA). The crystallinity χ_c,H_ of the POM samples was calculated from the heat of fusion according to Equation (1).

(1) $$\chi_{c,H}=\frac{{\Delta H}_{m}^{c}}{{\Delta H}_{m}^{0}}*100$$

The enthalpy of fusion of the sample determined by DSC was ${\Delta H}_{m}^{c}$. For the enthalpy of fusion ${\Delta H}_{m}^{0}$ of fully crystalline POM (100 % crystallinity), different values were reported. While often, an enthalpy of ${\Delta H}_{m}^{0}$= 317.93 J/g [49] was used, e.g., by [35], also enthalpies of 270 J/g and 326 J/g were reported [50]. Ehrenstein [50] gave an enthalpy of fusion of 220 J/g specifically for a POM copolymer, which was used in this work.

#### 2.3.7. Helium Pycnometry

Solid density was determined using a helium pycnometer AccuPyc 1330 (Micromeritics, Unterschleißheim, Germany) equipped with a 1 cm^3^ sample cell. The reported value was the average over 5 single determinations, with a relative standard deviation of about 1/1000. Due to the differing reported values of the enthalpy of fusion of fully crystalline POM, also solid density was used to evaluate and compare the crystallinities of the POM samples. The degree of crystallinity χ_c,d_ of the POM particles measured by pycnometry could be calculated according to Runt and Kanchanasopa [51] employing Equation (2):

(2) $$\chi_{c,d}=\frac{\left(\rho_{a}^{-1}-\rho^{-1}\right)}{\left(\rho_{a}^{-1}-\rho_{c}^{-1}\right)}*100$$

The density of the sample ρ was obtained via measurement; the density ρ_a_ of the amorphous and ρ_c_ of the fully crystalline POM were assumed as 1.215 g/cm^3^ and 1.492 g/cm^3^, respectively [52].

## 3. Results

### 3.1. Solvent Screening

Identification of potential moderate solvents for precipitation of POM, yielding micro particles being applicable in PBF, was done by screening experiments using autoclaves. For the preselection of promising solvents, several criteria were considered. First, the choice of potentially suitable solvents was made based on Hansen solubility parameters [53], which gave information on the interaction of the polymer with the solvent with respect to dispersion forces (cf. dispersive Hansen parameter, δ_d_), polar interactions (δ_p_), and hydrogen bond interactions (δ_h_). Based on these single contributions, the total Hansen parameter δ_t_ could be calculated. Generally, higher compatibility and, therefore, potential dissolution were indicated by a small difference of the total Hansen parameters Δδ_t_ of the polymer and the solvent. The sketched approach to find suitable solvents for the precipitation of PBF-suited polymer particles via the solution-dissolution process was reported previously [24,25,26,54]. For the screening study, only solvents with Δδ_t_ < 2 were considered. Furthermore, we ruled out solvents that were known to be good solvents that dissolved (semi-crystalline) POM at room temperature. Moreover, solvents were ruled out that were critical considering their hazardousness, toxicity, or their environmental impact [55]. In Table 1, the single Hansen parameters and the total Hansen parameters, as well as the difference in total Hansen parameters of POM and the chosen solvents used for screening are listed.

**Figure 1:**
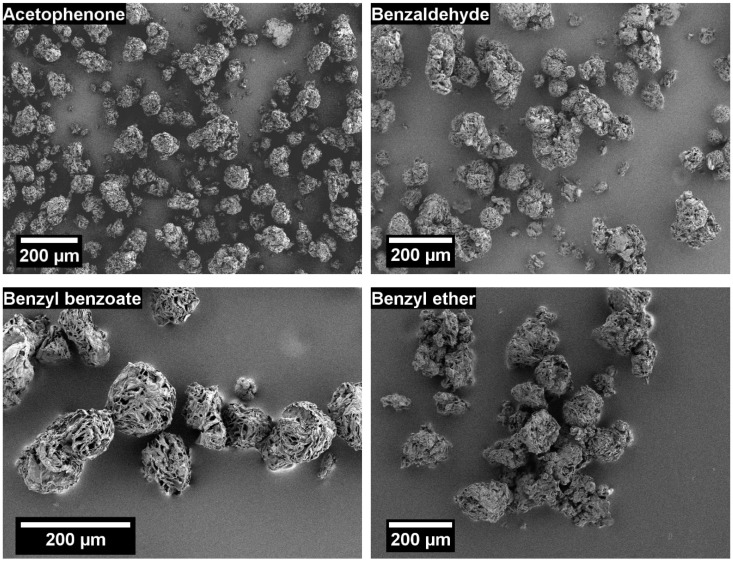

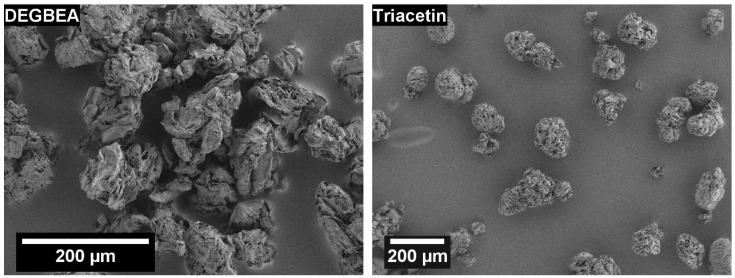
Scanning electron microscopy (SEM) images of precipitated Polyoxymethylene (POM) particles obtained in solvent screening experiments from acetophenone (top, left), benzaldehyde (top, right), benzyl benzoate (middle, left), benzyl ether (middle, right), diethylene glycol n-butyl ether aceta (DEGBEA) (bottom, left), and triacetin (bottom, right).

SEM images of the POM particles precipitated from the solvents used in the screening are depicted in Figure 1. Under the chosen conditions, non-spherical particles with a rough surface could be obtained by precipitation from each of the investigated solvents. Except for acetophenone and triacetin, besides the micro particles, also macroscopic “particles” or aggregates were observed. This was most significant for DEGBEA, benzyl benzoate, and benzyl ether, where a large solid POM piece in the shape of the bottom of the vessel was observed. This could suggest incomplete dissolution of the polymer, so that molten POM granules coalesced and sedimented to the bottom of the autoclave. Consequently, based on the observed yield of micro particles, acetophenone and triacetin were used for the further studies (see Section 3.2).

**Figure 2:**
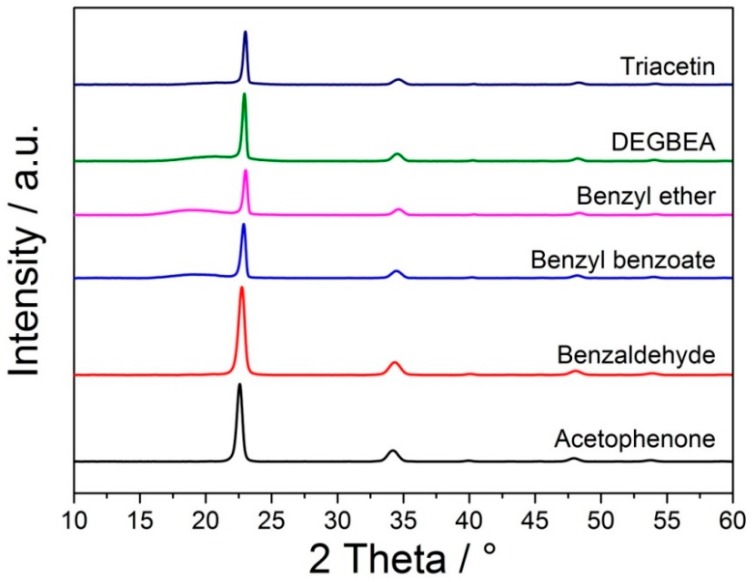
Diffractograms of POM powders precipitated from acetophenone, benzaldehyde, benzyl benzoate, benzyl ether, DEGBEA, and triacetin with an initial POM concentration of 10 wt.%.

Besides particle properties, especially the degree of crystallinity of the applied feedstock materials was essential for PBF processability and resulting part properties. Feedstock powders of semi-crystalline behavior typically showed good processability; a high contour accuracy could be achieved due to the specific melting range and sharp transition from solid to molten state during processing. To check qualitatively for crystallinity, the obtained POM particle samples were investigated by X-ray diffraction. Diffractograms of the precipitated powders are depicted in Figure 2. All samples showed the distinct reflexes indicating semi-crystalline hexagonal POM, with reflexes at around 23.25°, 34.75°, 40.55°, 48.50°, and 54.30° for the {100}, {105}, {111}, {115}, and {205} lattice planes, respectively [49]. Interestingly, an amorphous halo at around 21.80° [49] could be observed with increasing intensity for the POM particles obtained from triacetin, benzyl benzoate, DEGBEA, and benzyl ether. These halos, which appeared as broad reflexes with low intensity close to the strong reflex at 23.25°, suggested lowered degrees of crystallinity. For the powder precipitated from triacetin, the halo was very faint, but still visible. While XRD of POM powder obtained from benzaldehyde, similar to acetophenone, showed no halo, the incomplete dissolution observed in the screening experiments rendered it impractical. Thus, also with respect to high degrees of crystallinity, acetophenone and triacetin were the most promising solvents, which were considered for further development of a POM PBF powder.

### 3.2. Manufacturing of POM Feedstock for Powder Bed Fusion

#### 3.2.1. POM-Acetophenone and POM-Triacetin Cloud Points

**Figure 3:**
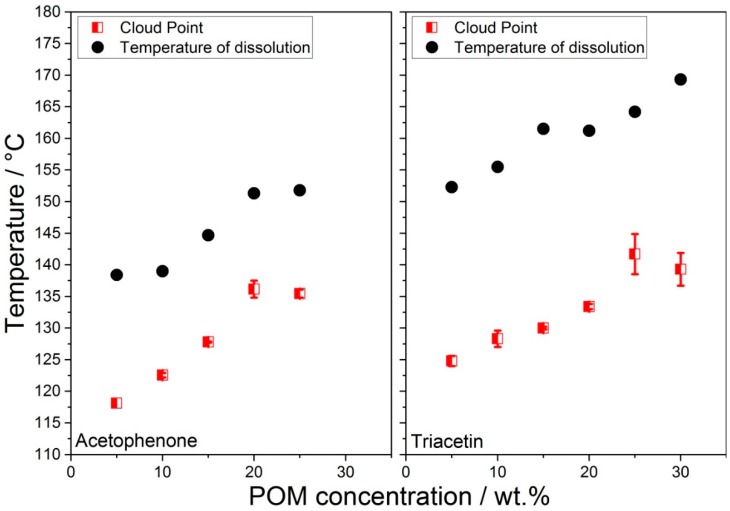
Cloud points of POM in acetophenone (left) and triacetin (right) for POM concentrations up to 30 wt.%. For the acetophenone system, investigation of the cloud point at 30 wt.% could not be achieved, due to incomplete dissolution.

For the further process development, a deeper understanding of the phase behavior of the considered polymer-solvent system was inevitable. Especially, knowledge about the dissolution temperature and the cloud point, i.e., the temperature where LLPS sets in, dependent on system composition, was necessary to deduce the appropriate process temperature profiles and process control. The advantage of the rather straightforward cloud point determination over the very cumbersome and tedious investigation of the full phase diagram (with miscibility gap, spinodal, and binodal) [56,57,58] was that it gave in an easy and fast measurement all the information necessary for process design. In Figure 3, the cloud-point diagrams for the POM-acetophenone (left) and POM-triacetin (right) systems are shown. The dissolution temperatures of POM in acetophenone were in the range between 138 °C and 152 °C for POM concentrations of 5 wt.% to 25 wt.%, while they ranged from 152 °C to 170 °C for POM concentrations of 5 wt.% to 30 wt.% in triacetin. An analogous trend could be observed for the cloud points, as they ranged from 118 °C to 136 °C for the acetophenone system and from 125 °C to 142 °C for the triacetin system. Typically, higher polymer concentrations led to higher cloud point temperatures, as, e.g., observed for polycarbonate in cyclohexanol [23] or poly(L-lactide) in triacetin [25]. For an ideal binary phase diagram, this trend would continue until the critical point, i.e., the point where binodal and spinodal coincided, was reached [36]. However, Rehage et al. [56] could show for a polystyrene-cyclohexane system that the phase diagram for technical polymers in moderate solvents was distorted and broadened, e.g., the critical point was shifted and not located at the maxima of the normally parabolic binodal and spinodal, due to the broad molecular weight distribution, rendering the system not a binary, but a multi-component system with each molecular weight fraction acting as a component. The lower temperatures of dissolution and the relatively smaller differences between dissolution and cloud point could be explained with the higher compatibility of acetophenone towards POM compared to triacetin, which was reflected in the smaller difference of total Hansen parameter Δδ_t_. Higher compatibility led to lower temperatures of dissolution, but also to smaller miscibility gaps [36,37,59].

#### 3.2.2. Effect of POM Concentration on Particle Shape

**Figure 4:**
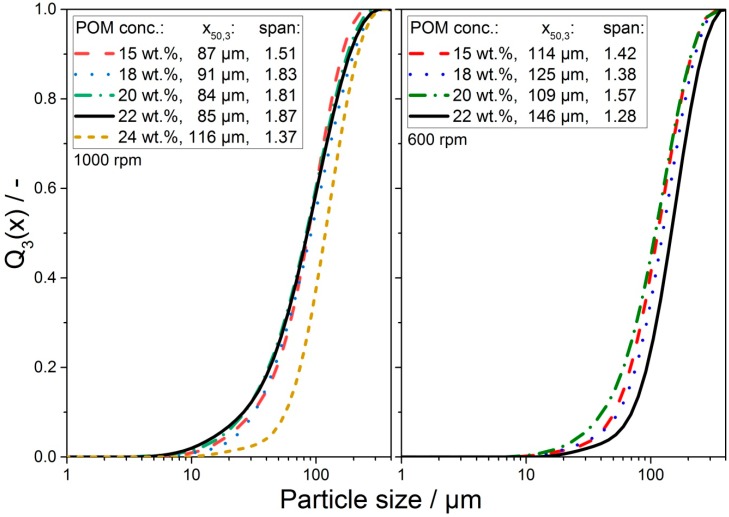
Volume weighted cumulative particle size distributions of POM particles precipitated from acetophenone (left) at 1000 rpm and triacetin (right) at 600 rpm with POM concentrations varying between 15 wt.% and 24 wt.%. Additionally given are the mean particle size x_50,3_ and the span.

**Figure A1:**
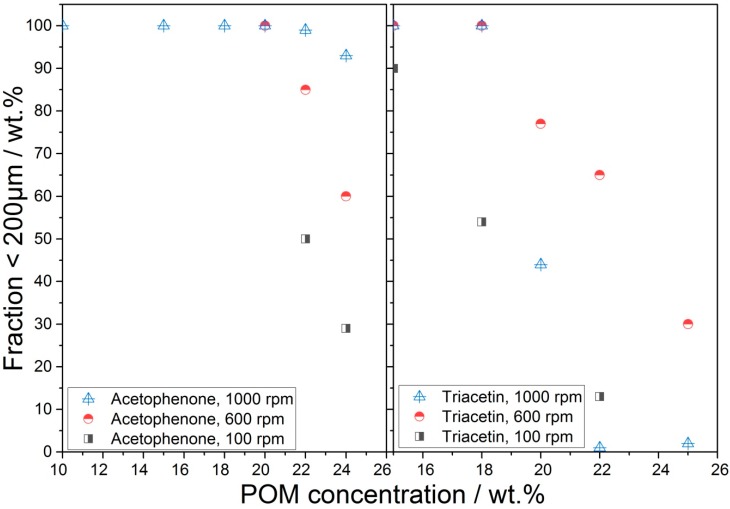
Yield of the particle fraction smaller than 200 µm for POM powders obtained from acetophenone (left) and triacetin (right) for varying initial POM concentrations and stirring speeds of 100 rpm, 600 rpm, and 1000 rpm. Since higher POM concentrations increased the overall yield of feedstock material, we aimed at high initial POM concentrations. However, if the formed particles became too large, application in PBF was impractical. Therefore, a cut size of 200 µm was chosen. Depending on the stirring conditions, the yield of particles below 200 µm varied greatly. Based on this evaluation, 20 wt.% and 22 wt.% POM in acetophenone with a stirring speed of 1000 rpm appeared favorable. For triacetin, the yield for 18 wt.% was nearly 100% for 1000 rpm and 600 rpm, while for 20 wt.%, it was already below 80% for 600 rpm and even below 50% for 1000 rpm. Consequently, POM concentrations of 18 wt.% and 20 wt.% with a stirring speed of 600 rpm were selected for further development.

Based on the knowledge of the cloud points, which elucidated the thermal boundary conditions, further investigation of the process parameters (i.e., POM concentration, stirring) on product properties was conducted. In preliminary parameter studies investigating the influence of stirring on the obtained POM particle size (please refer to Figure A1), a stirring speed of 1000 rpm for the acetophenone system and 600 rpm for the triacetin system was chosen. An important parameter in the evaluation of stirring speeds other than the obtained PSD was the fraction of product particles passing through a 200 µm sieve, as too large particles were problematic in the PBF process with powder spreading units typically optimized for particles in the size range of 50 to 70 microns (x_50,3_). In Figure 4, the PSDs given as the volume weighted cumulative particle size distribution Q_3_(x) of POM particles obtained via the solution-dissolution process with varying POM concentrations in acetophenone (left) and triacetin (right) are depicted. POM concentrations higher than 22 wt.% in triacetin led to very large particles for all investigated stirring speeds, which rendered them unemployable for PBF (cf. Figure A1). Therefore, we refrained from analyzing these samples via laser diffraction. Furthermore, the mean particle size x_50,3_ and the span, calculated as (x_90,3_-x_10,3_)/x_50,3_, indicating the distribution width, is given. It is clearly visible that the particles obtained from triacetin were generally larger for the same POM concentration than those obtained from acetophenone. The sizes of the POM particles precipitated from acetophenone were comparable to those of the polyamide 11 (PA11) particles manufactured via the solution-dissolution process from ethanol [40]. While, in principle, higher polymer concentrations led to larger particles and higher stirring intensity led to smaller particles, the overall interplay of LLPS droplet growth kinetics, supersaturation, system composition dependent initial droplet volume fraction, viscosity, temperature, droplet coalescence and ripening, and shear induced droplet breakup were involved and were not further addressed within this contribution. A comprehensive discussion of these dependencies can be found elsewhere; see, e.g., [36,37,38,39,40,57,58,60]. In short, higher initial polymer concentrations led to larger particles, as the volume fraction of polymer rich droplets increased with polymer concentration. This led to increased coalescence and Ostwald ripening, resulting in larger droplets and, subsequently, larger precipitated particles. Furthermore, also the collision probability of droplets was increased. Depending on the shear forces acting on the droplets induced by stirring, droplet breakup in droplet collision dominated. However, as more dissolved polymer increased the viscosity of the solution, shear induced droplet breakup might be hindered due to viscous damping. Subsequently, larger particles would be obtained from systems with higher polymer concentration. However, the exact implications of the solvent-polymer pairing on the obtainable particle sizes are not yet fully understood.

Compared to the cryo-milled and classified (x_max_ = 120 µm) POM copolymer powder used in [35] with 28 µm, 66 µm, 120 µm, and 1.39 for the x_10,3_, x_50,3_, x_90,3_, and span, respectively, the particles obtained by precipitation were larger and broader (acetophenone) or similarly (triacetin) distributed. Under consideration of the PSD and the particle yield, i.e., the fraction < 200 µm, products obtained using system compositions of 20 wt.% and 22 wt.% POM in acetophenone and 18 wt.% and 20 wt.% in triacetin were further investigated concerning their thermal properties (cf. Figure A1 for detailed information).

#### 3.2.3. Thermal Properties and Crystallinity of the Manufactured POM Powders

**Figure 5:**
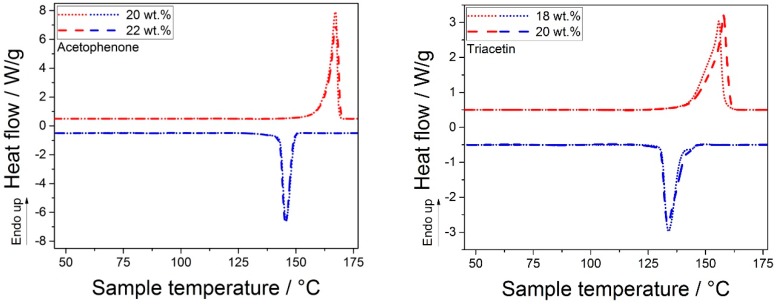
Thermograms of POM particles precipitated from acetophenone (left) and triacetin (right).

The thermal properties, especially the process window given as the temperature difference between the onset of melting and the onset of crystallization determined at a scanning speed of 10 K/min, were of utmost importance for the PBF process, as these defined the building chamber temperature in PBF. Small process windows required very precise temperature control in the building chamber and a homogeneous temperature profile, which was often difficult to ensure. In Figure 5, the thermograms of the POM samples obtained from acetophenone (left) and triacetin (right) are displayed. It can be observed that the samples obtained from triacetin showed broad melting endotherms and crystallization exotherms, which nearly overlapped. Consequently, the resulting process windows were too small, rendering the POM powders obtained from triacetin inapplicable in the PBF process. With processing windows of 2.6 K and 3.1 K for the 18 wt.% and 20 wt.% samples, the onsets of melting and crystallization were so close that minor changes in temperature, which could easily occur, e.g., in the building chamber or the powder bed, could lead to unwanted melting or premature crystallization. Accordingly, a robust process control was impossible, which could result in complete failure of the whole building job (cf. excessive melting of non-illuminated powder or extreme curling of the parts and subsequent part destruction or removal by the coater). On the contrary, the samples obtained from acetophenone showed distinctly separated melting exotherms and crystallization endotherms, resulting in process windows of 14.2 K and 14.6 K for the 20 wt.% and 22 wt.% samples, respectively.

In Table 2, the onsets of melting and crystallization, as well as the deduced process window and the degree of crystallinity of the samples calculated from the melting enthalpy and solid density, respectively, are listed. The samples obtained from triacetin showed, next to the small process windows, also degrees of crystallinity similar or even smaller than those found for the POM copolymer feed material. This result complemented the XRD pattern, where an amorphous halo could be observed for the samples precipitated from triacetin. On the contrary, the samples precipitated from acetophenone showed very high crystallinities, as confirmed by both measurement methods. Typically, particles obtained via the solution-dissolution process showed very high crystallinities, rendering this process beneficial for the manufacturing of PBF feedstock powders [23,24,25,40]. This was obvious, when comparing the crystallinity of the precipitated materials (see Table 2) to that of the cryo-milled POM copolymer used in [35], where a fusion enthalpy based degree of crystallinity of 42.3 % was reported (using a fusion enthalpy of 317.93 J/g for the perfect crystal), which corresponded to 61.2 % using the fusion enthalpy from the literature [50] employed in this work.

While the process window of 14.6 K observed for the favorable 22 wt.% POM in the acetophenone sample was rather small compared to 27.2 K and 31.8 K for commercial PA12 [8] and commercial PA11 [40], respectively, it was somewhat larger than the process window of 11.1 K reported for the cryo-milled POM copolymer applied to PBF in [35]. Therefore, the process window should be sufficient for PBF application.

#### 3.2.4. Product Powder Characterization

**Figure 6:**
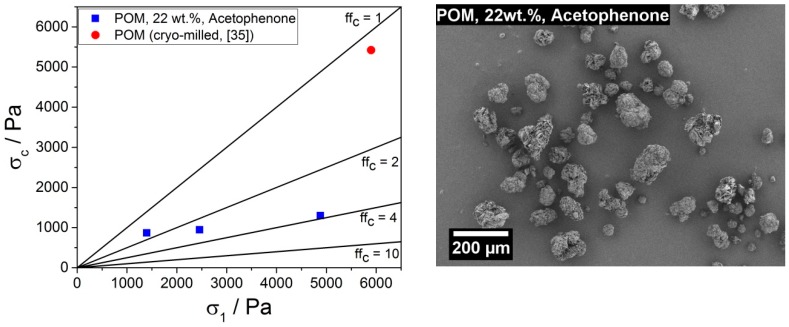
Left: Flowability ff_c_, as determined with ring shear experiments, of the manufactured POM PBF feedstock (blue squares) in comparison with the cryo-milled POM reference (red circle). Right: SEM image of the precipitated POM particles.

Based on the results concerning PSD and thermal properties, a larger batch (approximately 1 kg) of POM PBF feedstock was prepared by precipitation from acetophenone for a system with a POM concentration of 22 wt.% at a stirring speed of 1000 rpm. The powder flowability of this material was assessed by shear experiments using a ring shear tester. The obtained flow functions ff_c_, depicted in Figure 6 (left), were 1.6, 2.6, and 3.8 for major consolidation stresses σ_1_ of 1397 Pa, 2459 Pa, and 4880 Pa, respectively. These flow functions indicated a cohesive behavior according to Jenike [47]; materials with a ff_c_ between 1 and 2 are considered as very cohesive, between 2 and 4 as cohesive, between 4 and 10 as easy flowing, and above 10 as free flowing. The observed flowability was well comparable to a PA11 powder manufactured via the solution-dissolution process, which exhibited flow functions of 2.1, 2.9, and 3.7 for σ_1_ of 1405 Pa, 2572 Pa, and 4803 Pa, respectively. These flow functions were below the flowability of commercial PBF feedstocks [40], which mostly reach into the easy flowing range (cf. commercial PA11 feedstock PA1101 (EOS) with 2.1, 4.5, and 6.0 for σ_1_ of 1397 Pa, 2845 Pa, and 5273 Pa, respectively). However, commercial PBF powders (e.g., PA12 and PA11) typically were dry coated with nanoparticles [5,11,40,61,62], which acted as flowability enhancers [63,64]. The non-dry coated cryo-milled POM powder studied in [35] showed very cohesive behavior (cf. Figure 6 (left)) expressed by a ff_c_ of 1.1 at a σ_1_ of 5899 Pa, which probably was caused by the overall smaller particle size combined with the inevitable small (cohesive) dust fraction created during cryo-milling, as observable in the SEM images presented in [34,35].

Furthermore, we investigated the Hausner ratio as an additional indicator for flowability. With a Hausner ratio of 1.13 ± 0.01, obtained from a bulk density of (0.314 ± 0.003) g/cm^3^ and a tapped density of (0.356 ± 0.006) g/cm^3^, the flowability of the manufactured POM powder was considered “good” [65]. Since the particles displayed in Figure 6 (right) exhibited a rough surface and in some cases an open lamellar structure, to check for possible porosity, gas sorption measurements were performed. The determined mass-specific BET surface area of 3.8 m^2^/g did not indicate pronounced porosity of the POM powder. The observed mass-specific surface area was comparable to that of a commercial PA12 PBF feedstock (PA2200, EOS, 5.1 m^2^/g) produced via the solution-dissolution process, while a PA11 PBF feedstock (PA1101, EOS) manufactured via cryo-milling showed a smaller mass-specific surface area of 1.5 m^2^/g [40].

Under the consideration of the thermal properties, especially the small process window, and the flowability data, no dry coating with flowability enhancers (e.g., fumed silica) was performed prior to usage of the material in PBF. We wanted to omit any possible negative influence of the flowability enhancers (e.g., by acting as nucleating agents) on the process window and also deemed the flowability as already suitable for PBF.

#### 3.2.5. Application in the PBF Process

**Figure 7:**
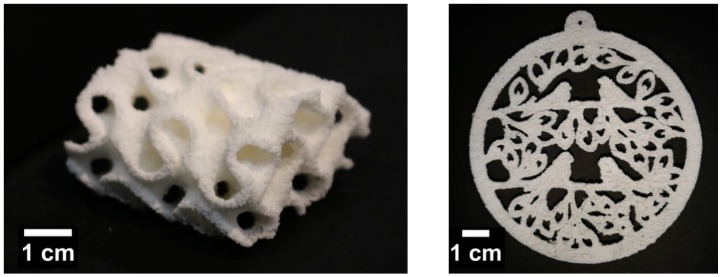
Test specimen printed from the developed POM feedstock powder. Left: gyroid. Right: ornament.

Before the precipitated POM powder could be employed in PBF, important processing parameters needed to be derived from the measured powder characteristics. For the manufacture of the desired parts, i.e., the gyroid and ornament specimens, their CAD files had to be processed with a slicer software that divided the parts into distinct layers resembling the spread powder layers in the PBF device. Based on the PSD, a layer height of 200 µm was set for slicing, using the freeware software “Slic3r” [66], which was recommended by the manufacturer of our desktop PBF machine. Furthermore, suitable building chamber temperatures could be derived from the assessed process window. In the SnowWhite device, temperature could be controlled by an “environmental temperature” measured by a sensor in the building chamber or the “powder bed temperature” measured close to the building platform. The powder bed temperature was approximately 20 K above the environmental temperature. For the processing of POM, powder bed temperatures in the range of 154–159 °C, corresponding to building chamber temperatures of 134–139 °C, were selected. Prior to the start of part manufacturing, a lag time of 15 min at the desired process temperature was selected, and additionally, eight warming layers were spread, to ensure thermal equilibrium. During processing, a lag time of 5 s was set between the end of sintering of one layer and the start of spreading of a new layer, in order to omit premature crystallization in the sintered layer, but also to mitigate overheating by too frequent laser illumination. For the selection of laser power and scanning speed, the recommended settings given by the SnowWhite manufacturer for commercial PA12 feedstock could be used for orientation, which were 2.8 W and 2188 mm/s for laser power and scanning speed, respectively. After a brief parameter study, a laser power of 2.8 W, a scanning speed of 1368 mm/s, and environmental temperatures of 134 °C and 136 °C for the gyroid and the ornament, respectively, could be identified to yield stable sintered parts. The difference in temperatures for the specimens stemmed most probably from the different geometries. The gyroid had much more volume, which corresponded to more thermal energy input by the laser, while on the other hand, the non-sintered powder in the inner pore structure of the gyroid should not be heated too much by the surrounding specimen borders. Thereby, baking of the non-sintered powder in the pores of the gyroid could be avoided, and the pores remained powder-free. The ornament had less layers, but a larger surface per layer, so that heat build-up in the part volume was not a problem, and accordingly, the building chamber temperature may be set 2 K higher. Examples of the manufactured gyroid and ornament are depicted in Figure 7.

The crystallinity of the manufactured parts was assessed to be around 58%, as determined via the solid density of cut segments of a gyroid, rendering the sintered parts slightly more crystalline than the injection molding feed material. While the parts showed a rough surface typical for non-post-processed PBF parts, they were stable and exhibited good detail precision and contour accuracy. The gyroid pores were free of powder, and the ornament details were well resolved, rendering the developed POM powder a suitable and applicable POM feedstock to manufacture POM parts via PBF.

## 4. Conclusions and Outlook

Starting from a solvent screening study to obtain particles from a bulk injection-molding POM copolymer grade, several moderate solvents yielding POM particles via LLPS and precipitation could be successfully identified via their solubility parameters. Acetophenone and triacetin were found as the most promising solvents, as they allowed for the highest polymer concentration to be processed in the precipitation. For both polymer solvents systems, appropriate production process parameters could be derived, which allowed manufacturing of POM particles in a PBF suitable size range. Supported by thermal characterization of the powders obtained from both solvents, further powder production was conducted solely with acetophenone, since the powders produced from this solvent showed a favorable process window of 14.6 K, being even larger than that of 11.1 K reported for the cryo-milled POM reference. The manufactured POM powder exhibited better flowability compared to the cryo-milled reference and was suitable for PBF, even without any flowability enhancers. Furthermore, the produced POM feedstock showed very high degrees of crystallinity of approximately 90% as determined via melt enthalpy and density, offering a sharp transition during PBF processing from solid to melt, which resulted in high detail precision and contour accuracy reflected in the manufactured gyroid and ornament specimens. While a comprehensive and successful workflow for the development of a POM PBF feedstock could be presented here, this study so far was limited to the demonstration of the principle feasibility of the proposed approach and the demonstration of basic PBF processability by means of a PBF desktop device. Upscaling of the process and a more detailed study on parameter dependencies like heating and/or cooling rate or isothermal precipitation, studies on further powder optimization, post-processing, or additive enhancement, as well as studies on PBF processability with industrial PBF machines and the manufacture of test rods including mechanical testing of these specimens will be the subject of future work.

## Figures and Tables

**Table 1:** Hansen solubility parameters of Polyoxymethylene (POM) and tested solvents [53]. DEGBEA, diethylene glycol n-butyl ether acetate.

Polymer/Solvent	δ_d_ (MPa^1/2^)	δ_p_ (MPa^1/2^)	δ_h_ (MPa^1/2^)	δ_t_ (MPa^1/2^)	Δδ_t_ (MPa^1/2^)
POM	17.1	3.1	10.7	20.41	—
Acetophenone	18.8	9	4	21.22	0.81
Benzaldehyde	19.4	7.4	5.3	21.43	1.02
Benzyl benzoate	20	5-1	5.2	21.28	0.87
Benzyl ether	19.6	3.4	5.2	20.56	0.15
DEGBEA	16	4.1	8.2	18.44	1.97
Triacetin	16.5	4.5	9.1	19.37	1.04

**Table 2:** Onsets of melting T_ons,m_ and crystallization T_ons,c_, process window, and degree of crystallinity χ of the investigated POM samples.

Sample	T_ons,m_ (°C)	T_ons,c_ (°C)	Process Window (K)	Crystallinity $\;\chi_{c,H}\;(\%)$	Crystallinity $\;\chi_{c,d}\;(\%)$
Acetophenone					
20 wt.%	163.4	149.2	14.2	83.3	88.8
22 wt.%	163.4	148.8	14.6	91.9	89.2
Triacetin					
18 wt.%	142.6	140.0/145.9*	2.6/—*	40.4	37.0
20 wt.%	143.9	140.8	3.1	56.5	53.6

* Onset and corresponding process window, if the first small exotherm is considered.
